# A Dynamic Gastrointestinal Mucus Simulator for Biorelevant Assessment of Nutrient Mucopenetration: Effects of Mucus Purification, Calcium, and Soluble Fibers

**DOI:** 10.3390/foods15173084

**Published:** 2026-08-31

**Authors:** Zijin Qin, Lewis Fortner, Jiannan Feng, Toshifumi Udo, Gopinath Mummaleti, Fanbin Kong

**Affiliations:** 1 Department of Food Science and Technology, University of Georgia, Athens, GA 30602, USA; zq20739@uga.edu (Z.Q.);; 2 Lewis Fortner Machine, 10755 Duncan Br Rd, Cleveland, GA 30528, USA

**Keywords:** nanocellulose, mucus permeability, mucus purification, dynamic digestion model, nutrient absorption, bioavailability

## Abstract

Gastrointestinal mucus is the first barrier encountered by luminal nutrients, but conventional static models do not reproduce mucus deformation under dynamic luminal flow. Additionally, the effects of mucus purification and dietary components on mucus barrier function remain insufficiently characterized. To address these gaps, a dynamic gastrointestinal mucus simulator was developed to evaluate mucus permeability under continuous luminal flow and investigate the effects of mucus purification and dietary components. The results showed that the dynamic model produced an approximately twofold glucose permeability compared with the static model, while increasing luminal flow further enhanced glucose permeability. Phycocyanin exhibited approximately 10-fold lower permeability than glucose, demonstrating the sensitivity of the model to differences in molecular properties. Additionally, mucus purification may compromise its barrier function due to the loss of gel-forming components. Dietary calcium and fibers enhanced the mucus barrier through different mechanisms, with 10 mM calcium reducing phycocyanin permeability by approximately 42%, and 6% cellulose nanocrystals and 2% alginate reducing glucose permeability by approximately 79% and 37%, respectively, suggesting their potential for modulating nutrient mucopenetration. This study provides an effective tool for evaluating mucus permeability and highlights the mucus barrier as an underexplored pathway where dietary components may modulate nutrient mucopenetration.

## 1. Introduction

Nutrient absorption in the gastrointestinal (GI) tract is governed by the interplay of multiple mechanisms, including passive diffusion (through both the lumen and the mucosal membrane), active transport, convective flow, and local degradation. The overall nutrient absorption reflects the integration of these cross-sectional local processes along the intestine, resulting in net axial convection, dispersion, degradation, and absorption [1]. Although nutrient absorption occurs locally at specific sites, this process is regulated longitudinally with consideration of the dynamics of the mucus layer, the first barrier of the GI tract.

As luminal digesta transits along the intestine, convective transport and local hydrodynamic conditions continuously alter the interaction between nutrients and the mucus layer. During luminal flow, nutrients progressively contact the mucus layer, inducing adhesive interactions and potential penetration into the mucus network. The viscous nature of mucus can entrap food components, whereas its elastic response to luminal flow, together with flow-induced mechanical abrasion, may induce local deformation and partial detachment of the mucus layer, resulting in increased local permeability and longitudinal redistribution of luminal components entrapped within shed mucus. Consequently, the mucopenetration of nutrient compounds greatly influences their subsequent absorption, and the apparent permeability (P_app_) is used as a first approximation of this mucopenetration capability [2].

To evaluate mucosal permeability, static models such as the Transwell [3,4], Franz diffusion cell [5], and Ussing chamber [6] are commonly used. However, these models provide limited biorelevance due to the absence of mixing of luminal contents induced by intestinal flow. As a result, local accumulation of tested compounds, together with the insufficient mucus fluidity and shear erosion induced by luminal flow, may collectively contribute to the misestimation of permeability. Although animal studies can overcome this limitation, the concurrent incorporation of other confounding physiological factors and intra-animal variability substantially complicates data interpretation [7]. Therefore, there is a need to develop an in vitro dynamic model that integrates the mucus layer and GI luminal flow to effectively investigate the nutrient mucopenetration profile.

Besides GI dynamics, the selection of the mucus material is another important factor influencing the accuracy of permeability evaluation. Although fresh native mucus collected post-mortem is generally preferred for most studies due to its high biorelevance, the presence of impurities (e.g., food debris and digestive secretions) may interfere with chemical quantification [8]. Consequently, purification is often used as a mucus pretreatment step for permeability testing [9,10,11], although its effects on mucus structure and permeability remain unclear.

As a structurally heterogeneous hydrogel with unique surface chemistry, GI mucus can interact with food components through multiple mechanisms, consequently altering its permeability [8]. Dietary calcium and fiber are recognized as nutrients of public health concern by the USDA [12], as a significant proportion of the population fails to meet the recommended daily intake. Additionally, their actual intake levels exhibit substantial inter-population variability due to diverse dietary habits and inconsistent supplementation practices [13,14]. Such fluctuations in intake levels expose the intestinal mucus layer to varying ionic and rheological environments, affecting the mucus permeability of co-ingested nutrients and orally administered compounds. Although recent studies have indicated interactions between dietary components and mucin proteins [15,16], most of this work has focused on static, molecular-level interactions between polymers and mucin proteins, rather than on the dynamic conditions of flowing luminal chyme during gastrointestinal digestion. Furthermore, the influence of subtle changes in the mucus network structure on nutrient mucopenetration efficiency remains underexplored.

Cellulose nanocrystal (CNC) is an emerging ingredient with multiple potential food applications [17], whereas alginate has already been widely used and recognized as a safe (GRAS) polysaccharide additive. Both polysaccharides have been reported for multiple GI health benefits, including delayed gastric emptying [18], altered digestion kinetics [19,20], and modulation of colonic microbiota [21,22]. However, before reaching the epithelium, these fibers inevitably interact with the GI mucus layer and may influence nutrient mucopenetration. CNC and alginate also exhibit distinct viscoelastic behaviors, which may lead to different interactions with mucus and effects on permeability, although experimental evidence remains limited.

Therefore, the aim of this study is to develop a dynamic digestion model incorporating GI mucus to effectively evaluate nutrient permeability across the mucus layer. The effects of mucus purification, dietary calcium, and soluble fiber ingestion on mucus permeability were investigated using the model to understand impacts of mucus selection and dietary components on nutrient absorption. The findings from this study provide a biorelevant tool for nutrient permeability evaluations and offer a new mucus-based insight into the mechanisms through which dietary components influence nutrient absorption in the GI tract.

## 2. Materials and Methods

### 2.1. Chemicals and Reagents

Cellulose nanocrystals (CNC, 12.1 wt% in water, containing 0.94 wt% sulfur) were produced by the Nanocellulose Pilot Plant of the Forest Products Laboratory (Madison, WI, USA). Sodium alginate (AG) was purchased from bioWORLD (Dublin, OH, USA). D-glucose was purchased from Sigma-Aldrich Chemical Co. (St. Louis, MO, USA). Commercial phycocyanin derived from Arthrospira platensis was obtained from Naturegrail (manufactured by Fluxias GmbH, Horgenzell, Germany), with a reported purity index (A620/A280) of approximately 2.0, a total protein content of approximately 61%, and no declared additives or excipients [23,24]. D-glucose HK Assay Kit (K-GluHK-220A) was purchased from Megazyme (Bray, County Wicklow, Ireland). All other chemicals used in this study were of analytical grade.

### 2.2. Mucus Collection

Porcine intestinal mucus was collected from postmortem jejunal and ileal tissues made available at the University of Georgia Swine Research Unit following slaughter conducted independently of the present study. The research team had no involvement in animal procurement, housing, treatment, handling, or slaughter, and no procedures were performed on live animals for this study. Within 2 h postmortem, the intestinal segments were rinsed with cold normal saline to remove food debris. Mucus was carefully scraped from the mucosal surface using a sterile spatula in a cold room at 4 °C. The collected mucus was pooled to improve sample homogeneity and subsequently stored at −80 °C until further use. The same batch of pooled mucus was used throughout the study.

### 2.3. Model Development

**Figure 1:**
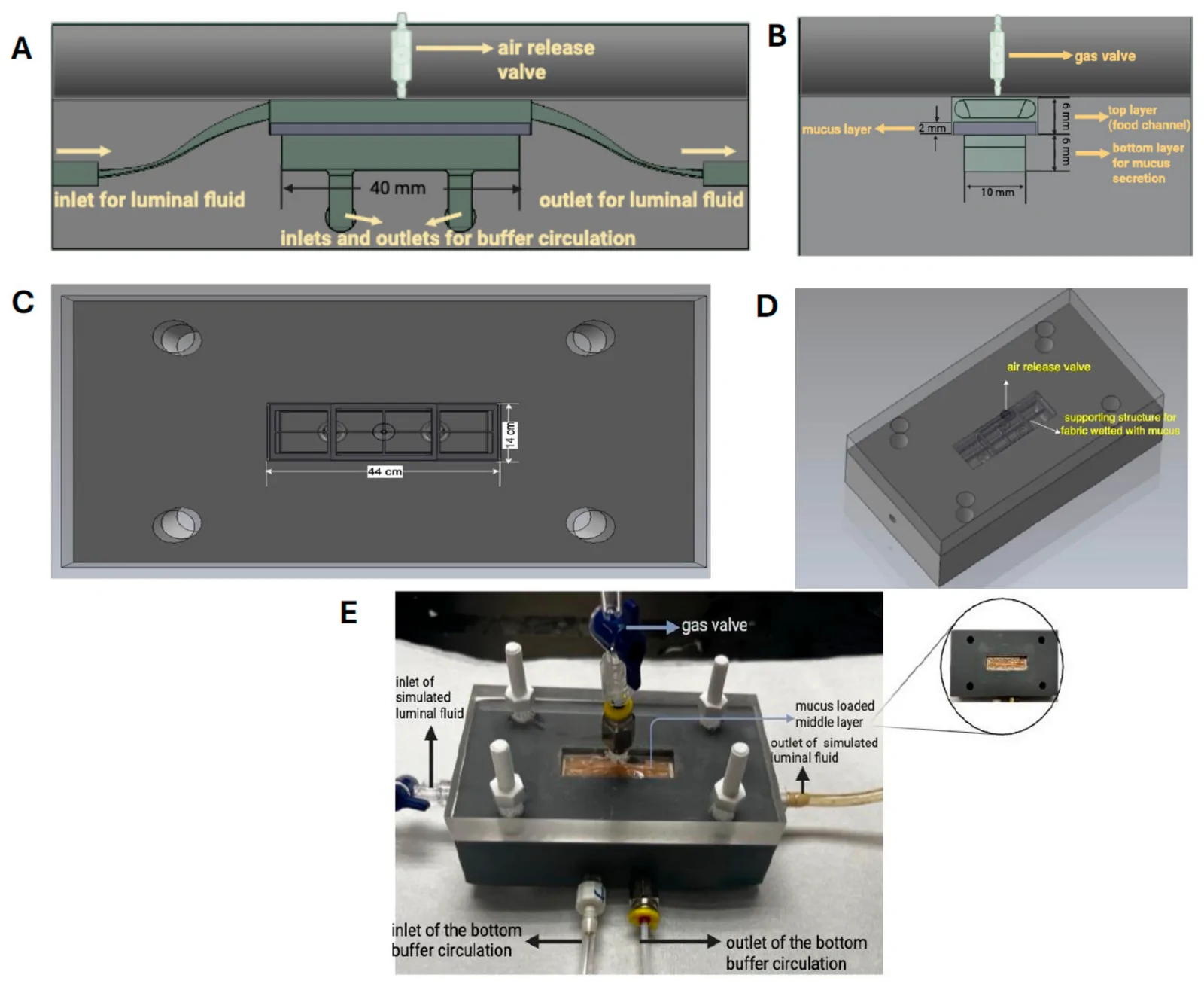
3D images of the Dynamic Gastrointestinal Mucus Simulator (DGMS): cross-sectional views along the XZ (**A**), YZ (**B**), and XY (**C**) planes; schematic illustration of overall structure (**D**); and actual photograph (**E**).

To evaluate the mucopenetration behavior of luminal nutrients during digestion, a Dynamic Gastrointestinal Mucus Simulator (DGMS) was developed by 3D printing (Figure 1). The model comprised three layers: the bottom layer was the reservoir for buffer solution, which circulated to mimic submucosal fluid. The middle layer functioned as the mucus compartment, where mucus was loaded onto filter paper, which provided structural support without inducing significant boundary effects due to its much larger pore size compared with the mucus mesh size. The top layer is a channel for chyme flow, where luminal flow imposed tangential shear stress on the mucus layer and induced erosion and washout of the mucus gel. Two tubes were connected to the inlets and outlets of the bottom and top chambers to allow the circulation of luminal and buffer solutions. A gas valve was embedded adjacent to the top layer to eliminate air bubbles that may interfere with mass transfer. Luminal flow rate and initial mucus thickness were set based on in vivo data [8,25,26,27]. This design allows the model to simulate nutrient mucopenetration under biorelevant scenarios, incorporating continuous hydration, erosion, and washout of the mucus layer, as well as the regulated flow of intestinal contents at adjustable rates, which are the crucial factors impacting the nutrients’ mucopenetration and adhesion [8]. Nutrients penetrating the mucus layer were continuously removed by the underlying buffer flow, preventing accumulation and the resulting underestimation of permeability commonly observed in static models [3,28].

### 2.4. Model Validation

Mucopenetration is influenced by both intrinsic permeability across the mucus barrier and the apparent disappearance behavior in the intestinal lumen. Intrinsic permeability is the inherent transport property of a solute across a barrier and is mainly determined by the physicochemical properties of the solute (luminal nutrients) and the barrier itself (mucus properties). In contrast, the apparent disappearance rate reflects the overall macroscopic transport behavior in the intestinal lumen and can be influenced by hydrodynamic conditions, such as luminal flow and mixing behavior [1].

Accordingly, the model was validated through three steps to assess both intrinsic permeability and apparent disappearance behavior. First, glucose permeability was evaluated at a fixed flow rate and compared with data from the static model to assess the necessity of incorporating luminal flow into mucus permeability assessment. Second, the permeability of luminal nutrients with different molecular sizes was examined under a fixed flow rate to verify whether the variation in intrinsic permeability was physiologically reasonable. Third, glucose permeability was evaluated under different flow rates to verify whether the permeability changes associated with hydrodynamic modulation of apparent disappearance behavior were physiologically reasonable.

#### 2.4.1. Principle of P_app_ Determination

P_app_ reflects the mucus barrier function and determines the nutrient absorption profile during digestion. It is derived from Fick’s law and the mass balance equation to evaluate the boundary effect between the donor and receiver sides for steady-state conditions [29]. The steady state occurs after an initial lag phase, during which cumulative glucose transport increases linearly with time, reflecting a constant mass transport rate across the mucus barrier.

In this study, the aqueous boundary layers on both sides, along with the filter paper and the mucus layer, collectively constituted the diffusion barriers. It has been previously reported that nutrient diffusion in the Side-Bi-Side diffusion system was not significantly affected by the two aqueous boundary layers [30]. Additionally, the resistance of the filter paper with a much larger pore size can be ignored [31]. Therefore, P_app_ (cm·s^−1^) in this study can be simplified as:

$$P_{app}=\frac{dQ}{dt}\times \frac{1}{A\times C_{0}}$$

where dQ/dt is the nutrients’ flux (mol·s^−1^) at the steady state; A is the surface area of the middle layer mucus (cm^2^); C_0_ is the initial concentration of the nutrient solution pumped into the top layer (mol·L^−1^), which was converted to mol·cm^−3^ during P_app_ calculation.

#### 2.4.2. Impact of Dynamic Luminal Flow on Mucus Permeability

**Figure 2:**
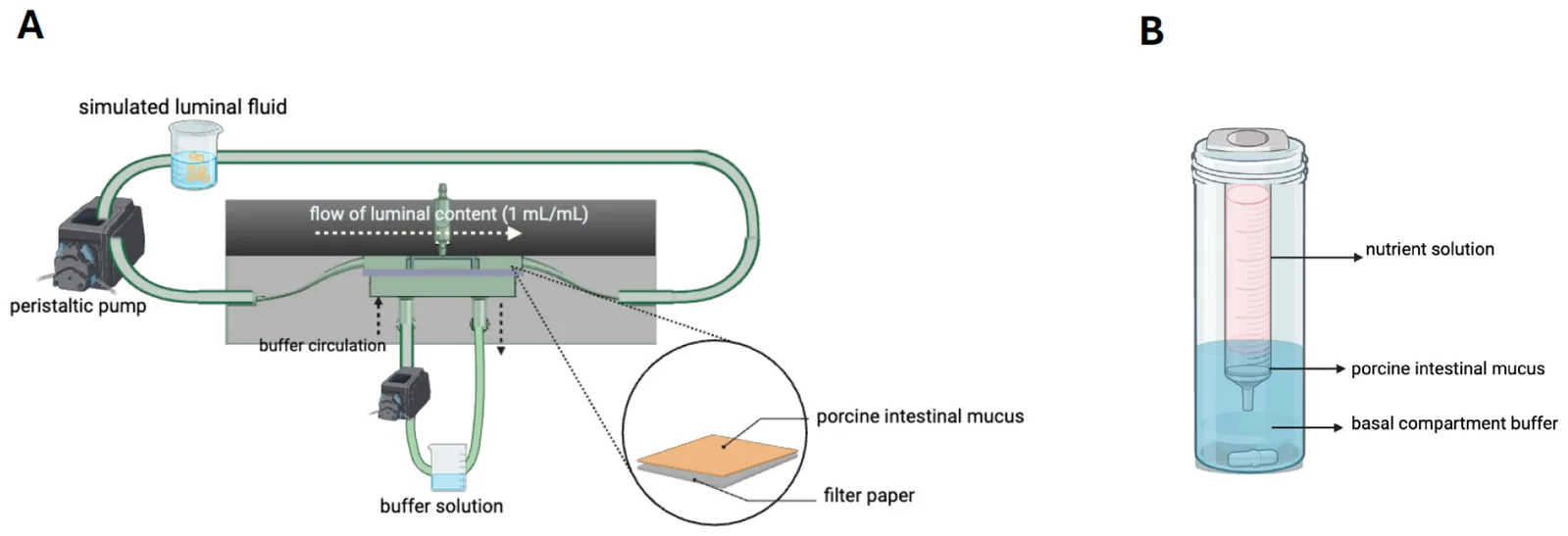
Set-up of the permeability test using the Dynamic Gastrointestinal Mucus Simulator (DGMS) (**A**); schematic illustration of the static mucus model (**B**). The figure was adapted from Qin et al. [8] with permission from the publisher.

To validate the necessity of incorporating dynamic luminal flow into mucus permeability evaluation, P_app_ was determined using both the DGMS and a static mucus model. D-glucose (40 g/L) was used as an example nutrient, and the resulting permeability values were compared with published data for model validation. The experimental setups of the static and DGMS models are shown in Figure 2.

For the DGMS (Figure 2A), mucus was carefully loaded onto the rectangular filter paper in the middle layer and evenly spread over an area of 3.24 cm^2^ to form a mucus layer with a thickness of 0.451 mm. The setup was held for 10 min to allow for mucus adhesion to the filter surface. Then, normal saline solution was pumped into the top layer at 1 mL/min to fill the apical cavity, and air bubbles were removed via the gas valve. The saline solution in the top and bottom layers was circulated for 10 min for system stabilization. Subsequently, the inlet solution in the top layer was replaced with 30 mL of simulated luminal solution containing the test nutrient at designated flow rates. The first 10 mL of the outlet fluid was discarded, followed by recirculation of the remaining top fluid. The bottom fluid was continuously recirculated throughout the experiment to maintain effective mass transfer. The entire system was enclosed in an acrylic hood with automatic temperature control, maintaining a constant temperature of 37 °C. Aliquots (500 μL) were collected from the bottom layer every 30 min. The collected samples were centrifuged at 14,000× *g* for 30 min, and the glucose concentration in the supernatant was determined using a glucose assay kit.

For the permeability test in the static model, a syringe mucus model was used according to previous literature [9] (Figure 2B). Mucopenetration was mainly driven by the concentration gradient and hydrostatic pressure. Briefly, a filter paper (D = 0.8 cm) was placed in a 3 mL syringe (ID = 8.66 mm). The syringe tip was submerged in a glass beaker (ID 14 mm, length 10 cm) containing a mini stir bar for mixing in the basal compartment. 5 mL of the receptor solution was loaded into the barrel and allowed to flow into the test tube to remove bubbles from the syringe tip. Native porcine mucus was then evenly loaded onto the filter paper, followed by the addition of 3 mL glucose solution into the syringe barrel. The thickness of the mucus in the static model was the same as that of the DGMS. The entire setup was maintained at 37 °C in an acrylic hood. Aliquots were collected from the basolateral compartment every 30 min, centrifuged to remove impurities, and the resulting supernatant was used for glucose quantification.

The cumulative amount of glucose transported to the bottom layer was plotted as a function of time (t) to determine the P_app_ under steady-state conditions. The steady-state flux (dQ/dt) was calculated from the slope of this linear region. Accordingly, the steady-state region was identified as 0.5–3.5 h for the static model and 1.0–3.5 h for DGMS, with R^2^ values above 0.98 and 0.99, respectively.

#### 2.4.3. Impact of the Molecular Size on Mucus Permeability

To evaluate the impact of nutrient molecular size on mucus permeability, glucose (40 g/L) and phycocyanin (200 g/L) were used as examples of small and large nutrient molecules for the permeability test. Mucus permeability was determined at a luminal flow rate of 1 mL/min using DGMS, and the mucus layer thickness was measured at the end of each trial. The steady-state flux was calculated from the slope of the linear region (R^2^ > 0.99) for glucose (1.0–3.5 h) and phycocyanin (1.0–4.0 h). The collected samples were centrifuged at 14,000× *g* for 30 min to remove large debris. Glucose concentration was quantified as described in Section 2.4.2. For phycocyanin quantification, absorbance was measured at 620 nm using a microplate reader (BioTek Synergy HTX, Winooski, VT, USA), and the phycocyanin concentration was calculated using a standard calibration curve within its established linear range.

#### 2.4.4. Impact of the Luminal Flow Rate on Glucose Mucopenetration

To evaluate the impact of flow rate on glucose mucus permeability, high (2.5 mL/min) and low (1 mL/min) flow rates were used for simulated top-layer fluid. The corresponding linear velocities (0.83–2.08 mm/s) were within the physiological range of small intestinal flow (0.15–2.5 mm/s) [32]. Mucus permeability and endpoint mucus thickness were determined through a permeability test. The steady-state flux of glucose (t from 1 h to 3.5 h) was calculated as the slope of the linear section of these curves (R^2^ above 0.97 for high flow rate; R^2^ above 0.99 for low flow mode).

### 2.5. Impact of Mucus Purification on Mucus Barrier Function

The DGMS was applied to evaluate the impact of mucus purification on mucus permeability. The permeabilities of native and purified mucus were compared in this section.

#### 2.5.1. Mucus Preparation

Native mucus was collected and used without further processing. For purification, mucus was diluted with cold normal saline at a ratio of 1:5, followed by well mixing and centrifugation. After removal of the supernatant and pelleted debris, the intermediate gel layer was collected and labelled as purified mucus [9,33].

#### 2.5.2. Impact of Purification on Mucus Permeability

To understand the impact of mucus purification on permeability, glucose mucopenetration was evaluated in the DGMS using different types of mucus. Steady-state flux was maintained at 1.0 to 3.5 h for native mucus and 0.5 to 1.5 h for purified mucus. Mucus thickness was measured after each trial using a vernier caliper.

#### 2.5.3. Impact of Mucus Purification on Mucus Rheology

Rheological analysis was conducted to further understand the impact of the purification process on mucus structure. HR-3 Discovery Hybrid Rheometer (TA Instruments, New Castle, DE, USA) equipped with a 1° cone angle and a 40 mm diameter plate was used for analysis. The plate temperature was maintained at 37 °C, and sample dehydration was prevented by applying low-viscosity mineral oil around the edges. A stress sweep test was first performed to determine the appropriate stress range within the linear viscoelastic region. This was followed by a frequency sweep (angular frequency 0.1–100 rad/s; oscillatory stress 2 Pa) and a steady-state flow test (shear rate 0.1–1000 s^−1^). Rheological parameters, including the elastic (storage, G′) and viscous (loss, G″) moduli as well as apparent viscosity, were obtained, and the complex viscosity (η*) was calculated according to the equation below, where ω is the angular frequency.

$$\eta *=\frac{{{(G}^{\prime })}^{2}+{{(G}^{\prime \prime })}^{2}}{\omega }$$

### 2.6. Impact of Dietary Calcium and Fiber on the Mucus Barrier Function

#### 2.6.1. Impact of Dietary Calcium and Fiber on Mucus Permeability

To evaluate the effect of calcium on mucus permeability, permeability tests were conducted using simulated intestinal fluid supplemented with 10 mM CaCl_2_, which was within the physiological range of luminal Ca^2+^ concentrations (5–20 mM) [3,34,35]. D-glucose (40 g/L) and phycocyanin (200 g/L) were used as examples of small and large molecules to investigate the impact of calcium on mucus permeability. Control groups without calcium addition were included for comparison. The steady-state flux was calculated from the slope of the linear region of the cumulative transport curves, with R^2^ values above 0.99 for the control groups and above 0.97 for the calcium-treated groups. At the end of the experiment, mucus thickness was also measured.

To evaluate the impact of dietary fibers on glucose permeability, CNC (6%) and alginate (2%) were selected as example fibers. The selected fiber concentrations were based on our previous study, which demonstrated digestive health benefits of CNC and alginate at these levels [18]. Additionally, these concentrations allowed each fiber system to exhibit its characteristic rheological behavior, thereby enabling comparison of mucus permeability between fibers with distinct rheological properties. Additionally, half-concentration samples (3% CNC and 1% alginate) were tested to examine concentration effects. The steady-state data from 1 to 3.5 h were used to calculate the slope of the linear region of each curve, with R^2^ values above 0.95 for the CNC group and above 0.99 for the alginate group.

#### 2.6.2. The Impact of Dietary Calcium and Fiber on Mucus Rheology

To further understand the interaction between different dietary components and mucus, rheological analysis was conducted. For sample preparation, three parts of native mucus were mixed with one part of CaCl_2_ solution, alginate, or CNC solution, respectively. The control group was prepared by adding an equivalent volume of deionized water. The samples were subjected to rheological analysis as described in Section 2.5.3. The mechanical moduli (G′ and G″) and apparent viscosity were recorded.

### 2.7. Statistical Analysis

All experiments were performed in triplicate using aliquots from the same pooled mucus batch, and the results were expressed as mean ± standard deviation. Statistical analyses were conducted by analysis of variance (ANOVA) followed by Tukey’s HSD test using JMP Pro 13.0.0 (SAS Institute Inc., Cary, NC, USA). Comparisons between two groups, including the effects of flow rate, mucus purification, and molecular size on mucus thickness and permeability, were analyzed using a two-tailed Student’s *t*-test. Statistical significance was set at *p* < 0.05.

## 3. Results and Discussion

### 3.1. Model Validation

In this study, model validation was conducted by examining permeability under various intrinsic factors, including nutrient molecular size and luminal flow rate.

#### 3.1.1. Influence of Luminal Dynamics on the Mucus Permeability Assessment

Although in vivo glucose absorption involves active transport across viable intestinal epithelium, such processes are hard to simulate in in vitro models. Effective permeability (P_eff_) is physiologically representative of overall intestinal absorption, particularly at luminal glucose concentrations below 250 mM, where transcellular transport predominates over paracellular transport [36]. However, determination of P_eff_ using ex vivo or in situ models is extremely complex, costly, and less suitable for high-throughput screening. More importantly, most P_eff_ testing models fail to readily distinguish the barrier contribution of mucus from that of the epithelial layer. Therefore, the P_app_ used in this study is a convenient and reliable first approximation of a compound’s ability to traverse the intestinal barrier [2].

The results of glucose permeability through the mucus layer in dynamic and static models are shown in Table 1. The P_app_ of the dynamic model was significantly higher than that of the static mucus model, which may be attributed to the enhanced mass transfer rate induced by the simulated luminal flow compared with conventional stir-bar mixing. During luminal flow, the mucus layer was subjected to shear stress, which not only induced internal flow and deformation of the mucus, but also enhanced convective mass transfer in the luminal cavity, collectively altering mucus layer thickness and nutrient permeability [37]. Additionally, the flow of the luminal fluid also reduced the thickness of the unstirred water layer (UWL) adjacent to the mucus layer. UWL is a relatively stagnant fluid layer adjacent to the mucosal surface, where solute transport occurs primarily by molecular diffusion [38]. In the healthy human jejunum in situ, its functional thickness is approximately 632 μm [39]. It is reported that improvements in luminal hydrodynamics can reduce this effective thickness [39]. Similarly, Wilson and Dietschy [40] also reported that stirring the bulk incubation solution during permeability tests reduced the UWL of the rat jejunum, leading to decreased Michaelis constants (K_m_) and increased permeability coefficients (P_app_) for the tested molecules.

In summary, by incorporating controllable luminal flow and the continuous removal of nutrients from the basal compartment, the DGMS shows greater physiological relevance than conventional static ones from model design and represents a promising tool for evaluating nutrient permeability across the mucus layer.

#### 3.1.2. Effect of Nutrient Molecular Size on Mucus Permeability

The results of the permeability of molecules with different sizes are shown in Table 2. The averaged P_app_ value of phycocyanin was around 10-fold lower than that of glucose, which was likely attributed to the enhanced meshing effect due to increased molecular size. Based on the classification proposed by Yee [41] and Walter et al. [42], intestinal compounds with permeability below 1 × 10^−6^ cm/s are regarded as low absorbable compounds. Although this threshold was originally derived from epithelial and mucus–epithelium co-culture models, the low P_app_ of phycocyanin observed across the isolated mucus layer in this study may partly explain its low bioavailability during GI transit. According to Stoll, Batycky, Leipold, Milstein and Edwards [1], molecular size strongly impacts nutrients’ intestinal permeability, as reflected by the corresponding membrane-scale Damköhler number (Da_m_). For example, ibuprofen (206 Da) exhibited high P_app_ (Da_m_ ≈ 1.6), while insulin (≈6000 Da) showed a very low Da_m_ (7.5 × 10^−4^), reflecting significant diffusion resistance within the mucosal layer.

Similarly, Fagerholm et al. [43] compared the permeability of various compounds in human jejunum in vivo and in the rat small intestine in situ and reported a molecular–size–dependent intestinal permeability for hydrophilic nutrients. As the molecular weight increased from approximately 60 Da (urea) to several thousand Da (PEG 4000), the permeability decreased markedly. A similar trend was also observed in computational simulations of the mucopenetration process, where particles with a diameter of 50 nm exhibited significantly enhanced mucopenetration compared with 200 nm particles [37]. While the observed glucose–phycocyanin difference in this study is consistent with this molecular-size-dependent trend, other physicochemical differences between the two compounds may also contribute to a lesser extent [44].

The comparable trend in P_app_ obtained from the DGMS using nutrients with different molecular sizes agrees with the current literature, supporting its biorelevance and applicability for evaluating luminal molecular permeability.

#### 3.1.3. Impact of the Luminal Flow Rate on Nutrient Mucopenetration

Luminal flow rate affects intestinal absorption by altering nutrient residence time, interfacial area between mucus and nutrients, and solute distribution within the chyme. The effect of flow rate on nutrient absorption is not unidirectional, depending on molecular size and the dominant transport mechanism. On one hand, for small molecules with high intrinsic mucus permeability, the high flow rate generally enhances recirculation and convective dispersion, thereby promoting mass transfer. On the other hand, for most macromolecules (e.g., insulin and calcitonin), increasing the flow rate does not substantially improve transmembrane diffusion because their transport is predominantly mucosal membrane-limited and less sensitive to GI hydrodynamics. Instead, the improved flow may shorten their local contact time with the mucus layer, thereby reducing bioavailability [1].

Accordingly, the transport mechanism of glucose in this study is predominantly diffusion-controlled, which is expected to be more susceptible to GI hydrodynamics than mucus barrier resistance. Consistent with previous literature, results in this study showed that increasing GI luminal flow rate significantly improved P_app_ value (Table 3), which may be attributed to the improved interfacial contact area, enhanced mixing, reduced UWL resistance, and increased intraluminal pressure at higher flow rates.

Besides the mixing effect, modulation of GI hydrodynamics may also influence mucus permeability through changes in mucus rheology. Increased luminal flow rate has been reported to weaken the entanglements and biointeraction between mucin fibers, thereby decreasing mucus viscosity during flow exposure [45]. At extremely high physiological shear rates, the viscosity of mucus gels can drop to levels comparable to water [46], potentially substantially enhancing molecular permeability. For example, in cervical mucus, reduced viscoelasticity during ovulation greatly improves the mucopenetration of sperm cells [46,47]. Similarly, in the GI environment, changes in luminal flow rate may alter nutrient permeability by modulating mucus microviscosity. Additionally, although not statistically significant (*p* = 0.3), the mucus thickness increased by approximately 9.3% when the flow rate decreased in this study. This change indicates that high flow rates may cause mild shear-induced erosion of the mucus layer, thereby affecting its steric barrier effect.

Collectively, the observed increase in glucose P_app_ under high flow rate was consistent with physiological trends and may be attributed to changes in mucus viscosity and steric barrier integrity within the DGMS. This agreement supports the physiological relevance of the system.

### 3.2. Influence of Mucus Purification on Permeability

To evaluate the feasibility of using purified mucus as an alternative source for permeability study, the permeability of purified and native mucus was compared using DGMS (Table 4). Purified mucus showed significantly higher permeability than native mucus. Both mucus types exhibited substantially increased thickness at the end of the trial compared with the initial loading height, likely due to hydration-induced swelling. Additionally, the purified mucus layer was relatively thinner at the end of the experiment compared with the native one (0.80 mm vs. 0.94 mm, *p* = 0.14), suggesting that it was more susceptible to erosion under luminal chyme flow. This is also consistent with the visual observations at the end of trials. The native mucus remained well hydrated and uniformly covered the supporting paper, forming an intact barrier for mass transfer, while purified mucus exhibited pronounced erosion and uneven surface coverage.

**Figure 3:**
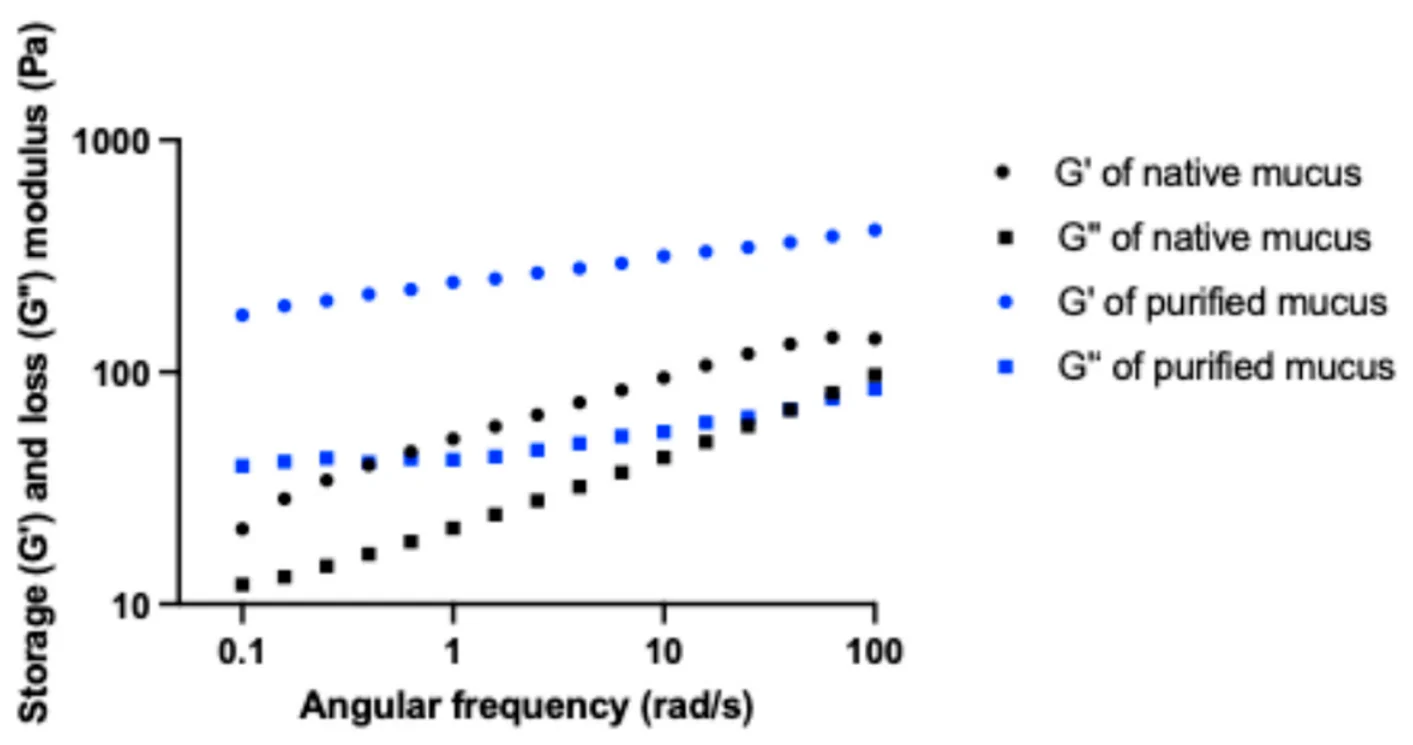
Storage modulus (G′) and loss modulus (G″) of native and purified mucus.

**Figure 4:**
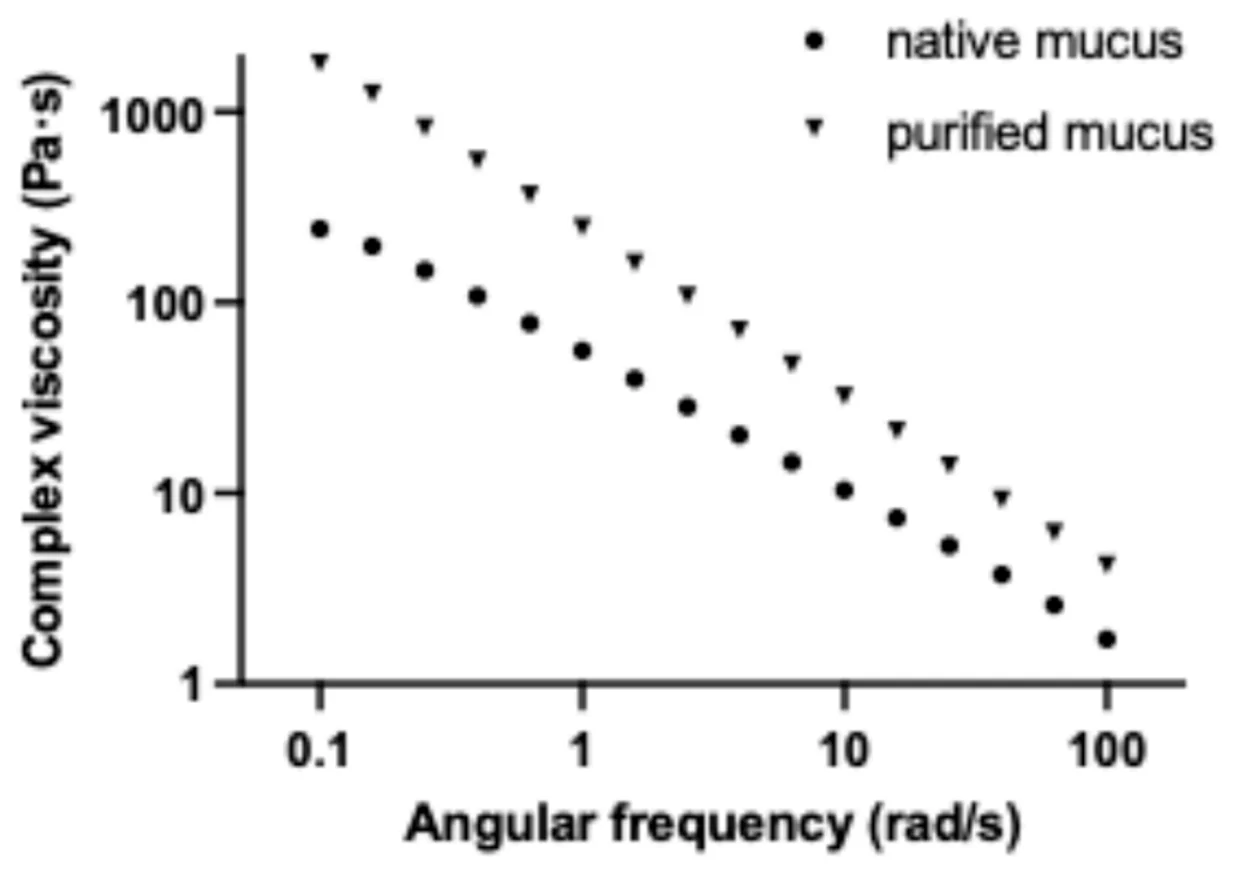
Complex viscosity of native and purified mucus.

**Figure 5:**
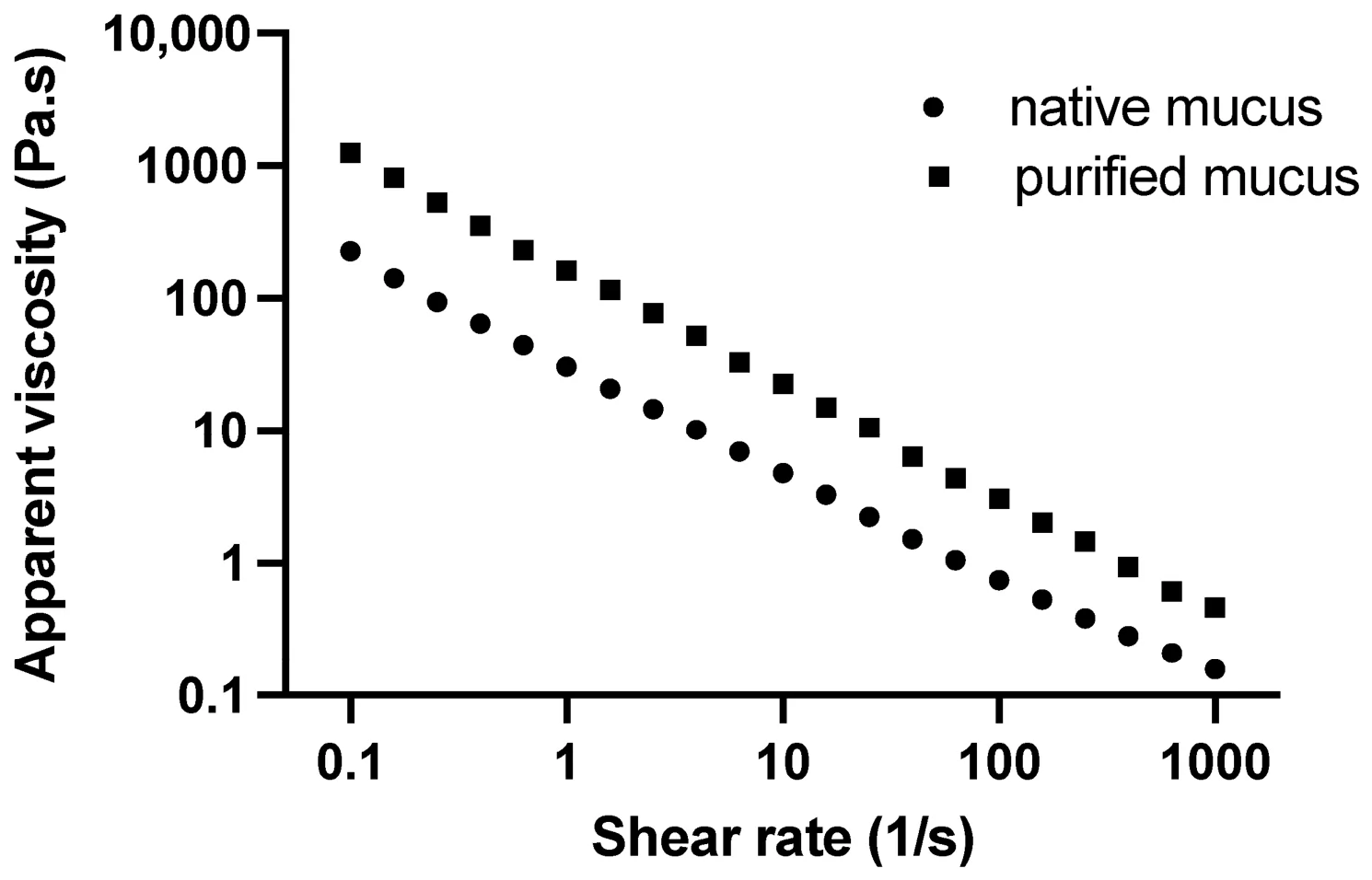
Apparent viscosity of native and purified mucus.

**Figure 6:**
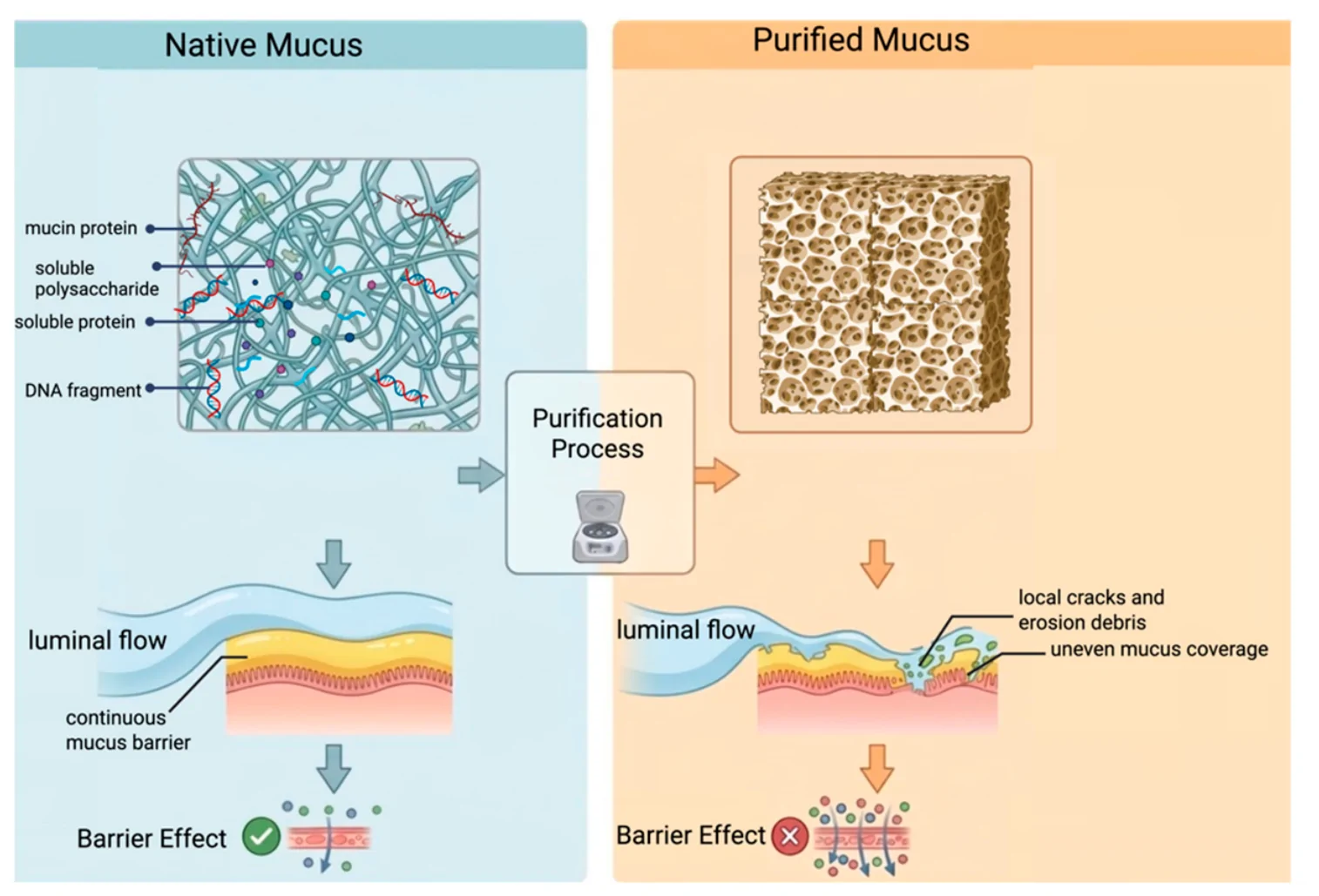
Schematic illustration of the proposed structural differences and barrier mechanisms between native and purified mucus. The figure was adapted from an image generated by Gemini.

The mechanical modulus, complex and apparent viscosity of the native and purified mucus are shown in Figure 3, Figure 4 and Figure 5. The purified mucus exhibited significantly higher storage modulus and apparent viscosity than native mucus, which may be attributed to compaction of the gel network during centrifugation in the purification process. The increased elasticity may promote the formation of local cracks and erosion debris under the frictional forces generated by continuous chyme flow, thereby disrupting the continuity and flexibility of the barrier structure covering the submucosal layer (proposed mechanism illustrated in Figure 6).

Although the previous numerical simulation reported that reduced mucus viscosity may decrease mucus flow resistance and increase permeability, these conclusions were based on simplified conditions, including homogeneous mucus properties and idealized boundary conditions [37]. In contrast, the DGMS, incorporating GI mucus and dynamic luminal flow, suggested that overall permeability cannot be explained simply by mucus viscosity but is also influenced by mucus barrier integrity and microstructure. Similarly, Sanders et al. [48] reported that increased viscoelasticity of sputum was associated with increased permeability of nanoparticles, which was attributed to a transformation of the gel network from a homogeneous microporous structure to a more heterogeneous macroporous one. A comparable phenomenon in biological gels may also occur in the present study, where mucus purification likely disrupted the original continuous mesh network and produced a more rigid, elastic, and heterogeneous structure with enlarged pores, thereby facilitating nutrient penetration during luminal flow.

Besides rheological performance, one possible mechanism underlying the observed reduction in mucus barrier function is that the purification process may remove key gel-stabilizing components. For example, discarding the diluted mucus supernatant during the washing step may lead to the loss of soluble gel-forming elements, such as MUC6, extracellular DNA, and their soluble fragments [49].

These components normally fill mucus pore spaces, increase chain entanglement, and contribute to the charge density of the network, and they have been reported to possess strong gel-forming capacity [49].

### 3.3. Impact of Dietary Calcium and Fiber on Mucus Permeability

#### 3.3.1. Dietary Calcium

**Figure 7:**
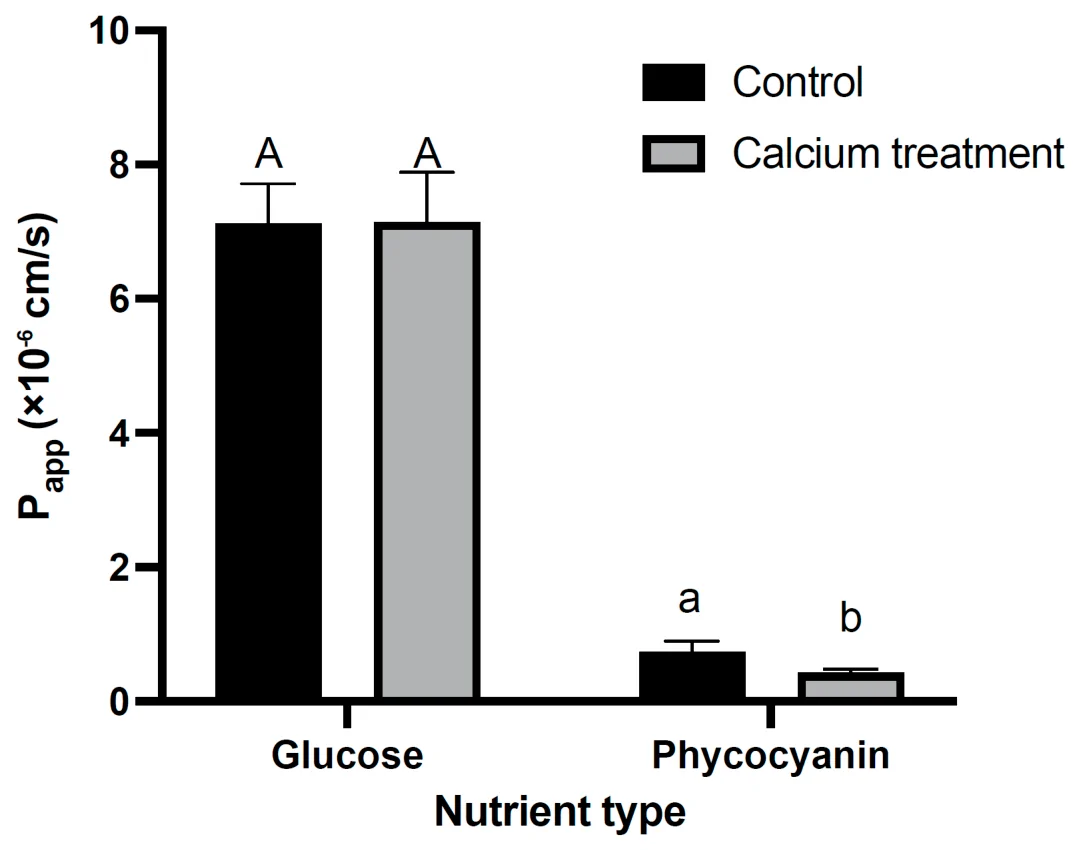
Impact of calcium treatment on nutrient mucus permeability; uppercase letters indicate statistical comparisons between control and calcium treatment for glucose; lowercase letters indicate statistical comparisons between control and calcium treatment for phycocyanin. Comparisons were made only within each nutrient (i.e., uppercase letters are not compared to lowercase letters). Different letters within the same case indicate a statistically significant difference (*p* < 0.05).

**Figure 8:**
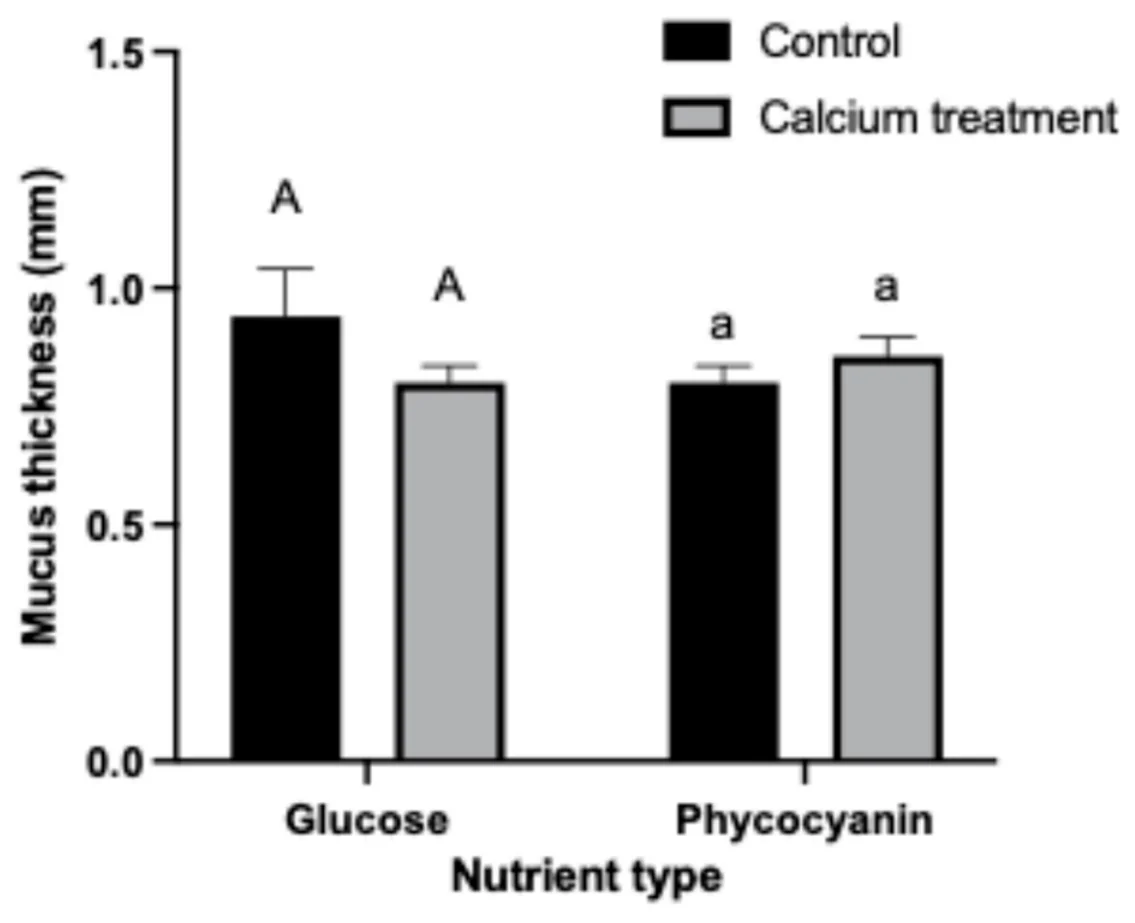
Impact of calcium treatment on mucus thickness. Uppercase letters indicate statistical comparisons between control and calcium treatment for glucose; lowercase letters indicate statistical comparisons between control and calcium treatment for phycocyanin. Comparisons were made only within each nutrient (i.e., uppercase letters are not compared to lowercase letters). Different letters within the same case indicate a statistically significant difference (*p* < 0.05).

The effects of calcium on mucus permeability and thickness for different molecules are shown in Figure 7 and Figure 8.

The incorporation of 10 mM calcium into the luminal fluid significantly decreased the P_app_ of phycocyanin by approximately 42%, likely due to the altered mucus meshing effect induced by the formation of complex mucin assemblies through calcium-mediated protein cross-links, although changes in ionic strength and osmolarity may also have a minor contribution to this effect [16]. Similarly, Sharma, Kwak, Kolewe, Schiffman, Forbes and Lee [3] reported that a dietary range of calcium (1–20 mM) promoted mucin aggregation by interacting with mucin glycan chains, thereby strengthening the mucus network, decreasing pore size, and reducing the permeability of large molecules (FITC–dextran, molecular weight of 20 kDa). However, a similar permeability-reducing effect of dietary calcium was not observed for glucose in this study, indicating its relatively lower sensitivity to the altered mucus network. As previously reported, extremely small luminal contents with sizes below the mucus mesh spacing can rapidly and freely penetrate GI mucus [46]. Consistent with this, in the present study, for small molecules such as glucose, whose molecular dimensions are substantially smaller than those of larger macromolecular nutrients such as phycocyanin, changes in pore size may have a relatively limited steric effect on diffusion.

Therefore, the insignificant effect of calcium on glucose permeability may be attributed to its remarkably small molecular size, allowing free diffusion through the cross-linked mucus mesh with less steric restriction. This finding also suggests the promising role of calcium in dietary strategies to modulate nutrient absorption and in the design of targeted mucus-based delivery systems.

The impact of dietary calcium on mucus thickness was not statistically significant by a two-tailed t-test. However, the calcium group showed approximately a 15% reduction in mucus thickness compared with the control, although this difference was not statistically significant (*p* = 0.1224). This trend may be attributed to the densification of the mucin network induced by calcium addition. Calcium-mediated crosslinking reinforced the mucin network, creating a dense structure with reduced hydration and swelling capacity. In contrast, the looser network of the control group, characterized by weaker intermolecular interactions, may allow for more extensive hydration and rapid expansion during luminal flow. Consistent with this interpretation, mucus collected at the conclusion of trials with calcium treatment appeared more opaque, with a matte, less hydrated surface compared to the control group (Figure S1). Similarly, Ermund et al. [50] also reported that reduced available calcium cations in mouse ileal mucus increased mucus flowability and weakened its attachment strength, suggesting the important role of luminal calcium in maintaining the morphology and structural integrity of the mucus network.

#### 3.3.2. Dietary Fiber

**Figure 9:**
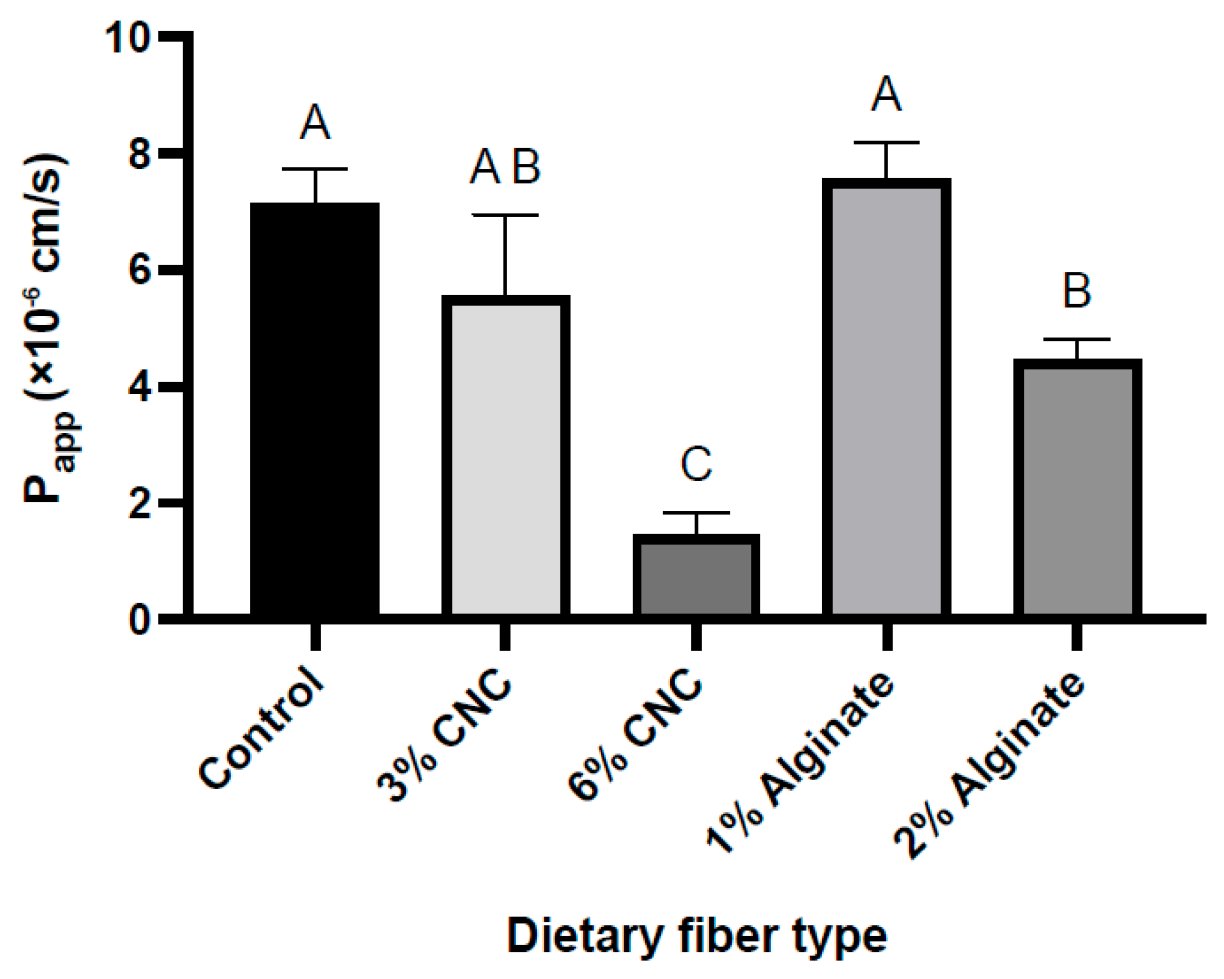
Impact of different dietary fibers on nutrient mucus permeability; different letters indicate the statistical difference (*p* < 0.05).

**Figure 10:**
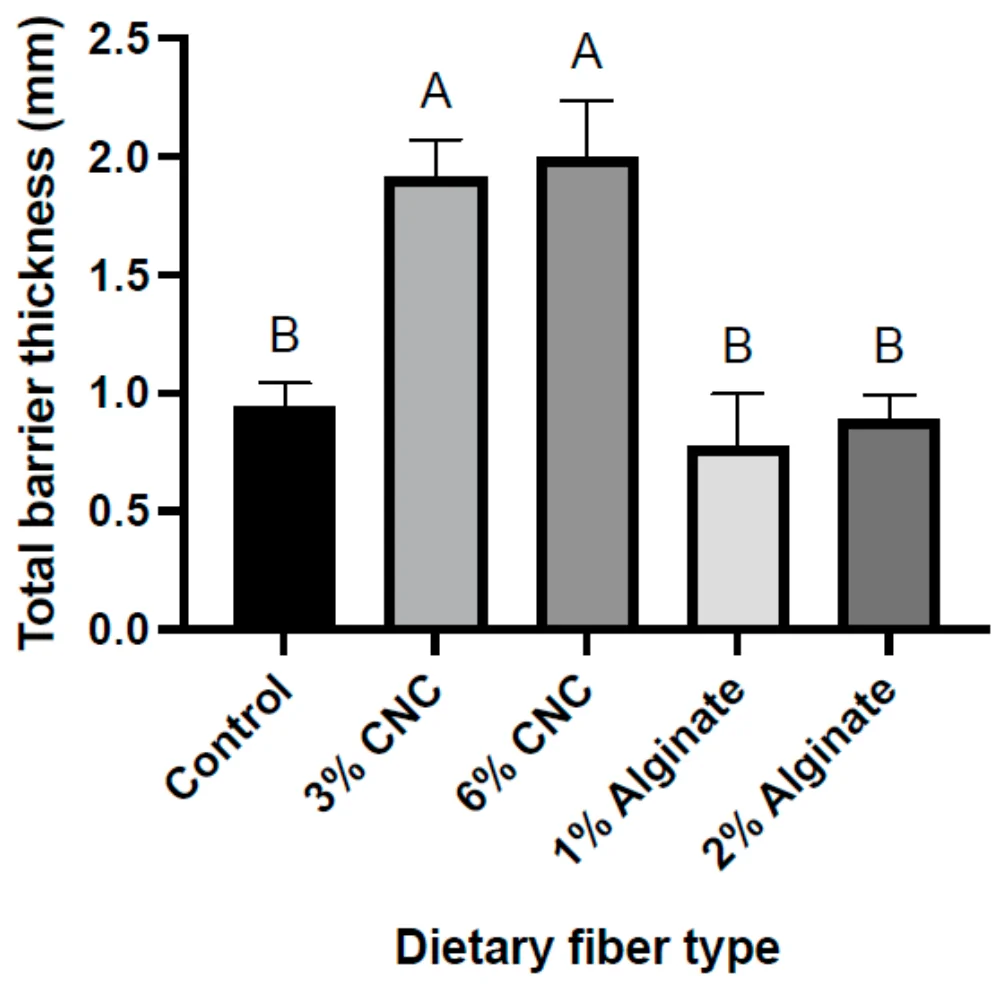
Impact of different dietary fibers on total barrier thickness; different letters indicate the statistical difference (*p* < 0.05).

The effects of dietary fibers on mucus permeability may vary according to their surface chemistry and physicochemical properties. For example, chitosan and hyaluronic acid were reported to enhance nutrient mucopenetration in the HT29-MTX cell model [51], while oat fiber was reported to reduce mucus permeability in an animal study [52]. The effects of CNC and alginate on mucus permeability and thickness are shown in Figure 9 and Figure 10. The incorporation of 6% CNC and 2% alginate significantly reduced mucus permeability by 79.4% and 37.4% compared with the control, indicating the marked contribution of these soluble fibers to mucosal barrier enhancement. At the end of the permeability test, the mucus in the alginate group exhibited an appearance similar to the control, while the CNC group exhibited a homogeneously spread hydrocolloidal layer adhering to the mucus surface, accompanied by a marked thickening of the mucus barrier (Figure S1). This distinct morphological difference suggests that the mechanisms underlying the barrier-enhancement effects may vary depending on the surface chemistries and physicochemical properties of the two dietary fibers.

Alginate is characterized as a non–mucus-interacting soluble fiber and alters mucus permeability mainly through physical modification of the mucus microenvironment. Mackie et al. [53] reported that alginate could freely diffuse into the mucus network without interacting with mucin protein. Even at a low concentration (0.1%), sodium alginate showed relatively high diffusion coefficients (10^−12^ to 10^−11^ m^2^/s), as quantified by fluorescence recovery after photobleaching (FRAP). The diffused alginate accumulated at the mucus boundary region and physically occluded local pores, thereby reducing mucopenetration of 500 nm probes. Correspondingly, in this study, the absence of any statistically significant change in mucus thickness after alginate treatment (*p* = 0.45) further supports the conclusion that alginate may enhance mucus barrier function by internal pore-filling instead of the thickening effect induced by adhesive interaction or surface accumulation.

In contrast, CNC is reported as a mucoadhesive fiber [31]. Although unable to penetrate the GI mucus layer, it modifies the physicochemical properties at the mucus surface and, through adhesive interactions, may increase steric hindrance to glucose penetration. Due to the abundance of hydroxyl groups and surface charge, this polysaccharide may form hydrogen bonds or electrostatic interactions with the mucosal surface or between polymer chains, forming aggregates that hinder nutrient permeation. This adhesive behavior is further enhanced by the nanoscale nature of CNC, which provides a high surface area for interaction with mucin proteins. Similarly, Hagesaether [51] reported that pectin reduced the mucosal P_app_ of carboxyfluorescein using the HT29-MTX cell model, which was attributed to its high molecular weight and abundant functional groups that promote biointeraction with the mucosal surface.

The mucoadhesive interaction of CNC was further evidenced by the significant increase in mucus thickness, which more than doubled compared with the control, indicating the formation of a substantial surface-associated hydrocolloidal barrier. Similarly, Lin, Shatkin and Kong [9] also reported the biointeraction of nanocellulose with a mucin solution, as reflected in improved gel structure, enhanced charge-based repulsion, and rheological synergism. The co-incubation of CNC with phenol red and fluorescein isothiocyanate–dextran 4 kDa (FD4) significantly reduced their mucopenetration in the static modified Franz diffusion cell model [31].

Additionally, alginate and CNC may reduce mucus permeability through mechanisms common to polysaccharide-based dietary fibers, such as compression of the mucus layer, entrapment of luminal nutrients within the fiber network, and thickening of the luminal contents and the UWL adjacent to the mucus [8]. For example, nanocellulose has been reported to retard glucose diffusion during upper GI digestion simulation and to reduce glucose uptake by the mucosal layer in a mucus-containing co-culture cell model [54,55]. The reduced glucose permeability induced by CNC and alginate suggests their potential role in modulating nutrient absorption, which may have implications for weight management. Furthermore, these properties support their application in mucus-responsive encapsulation and mucoadhesive drug delivery systems [56].

### 3.4. Impact of Dietary Calcium and Fiber on Mucus Rheology

**Figure 11:**
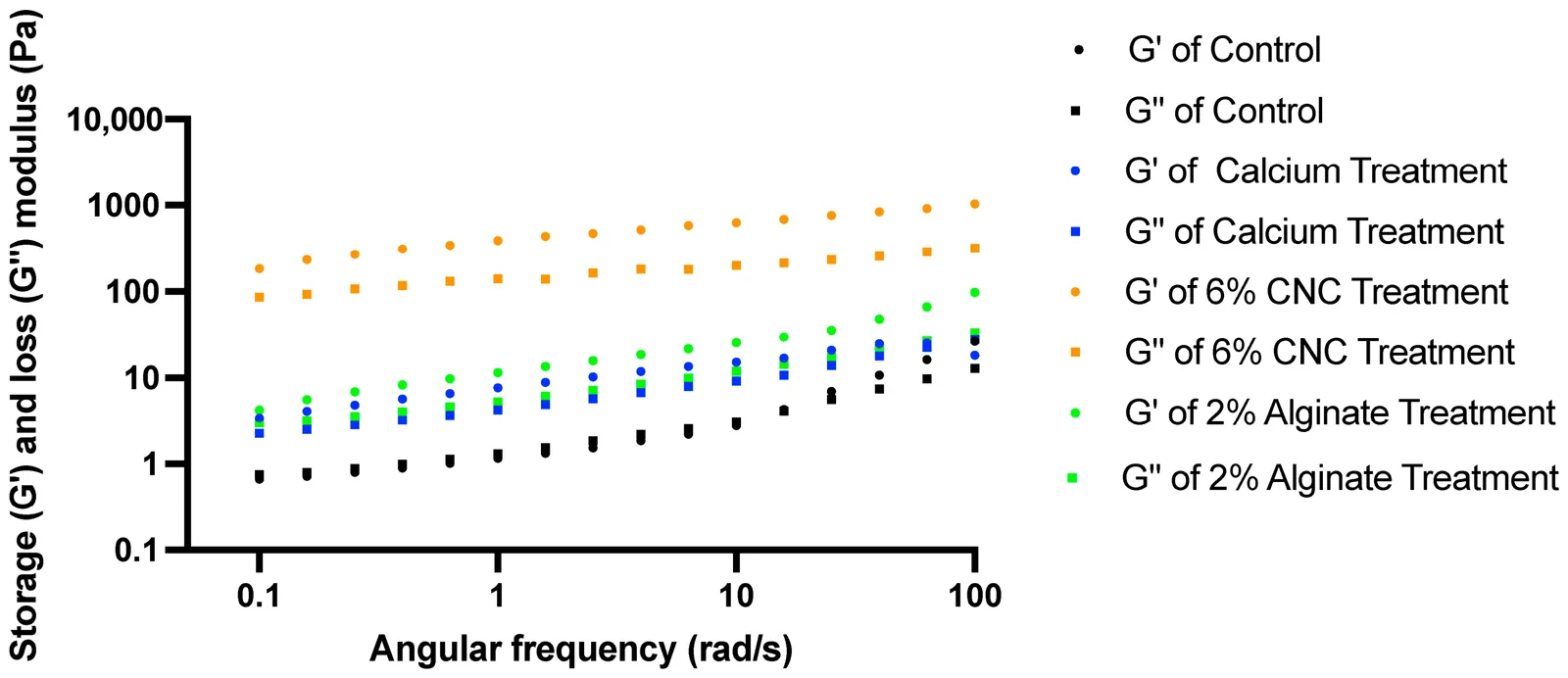
Storage modulus (G′) and loss modulus (G″) of mucus with various dietary treatments.

**Figure 12:**
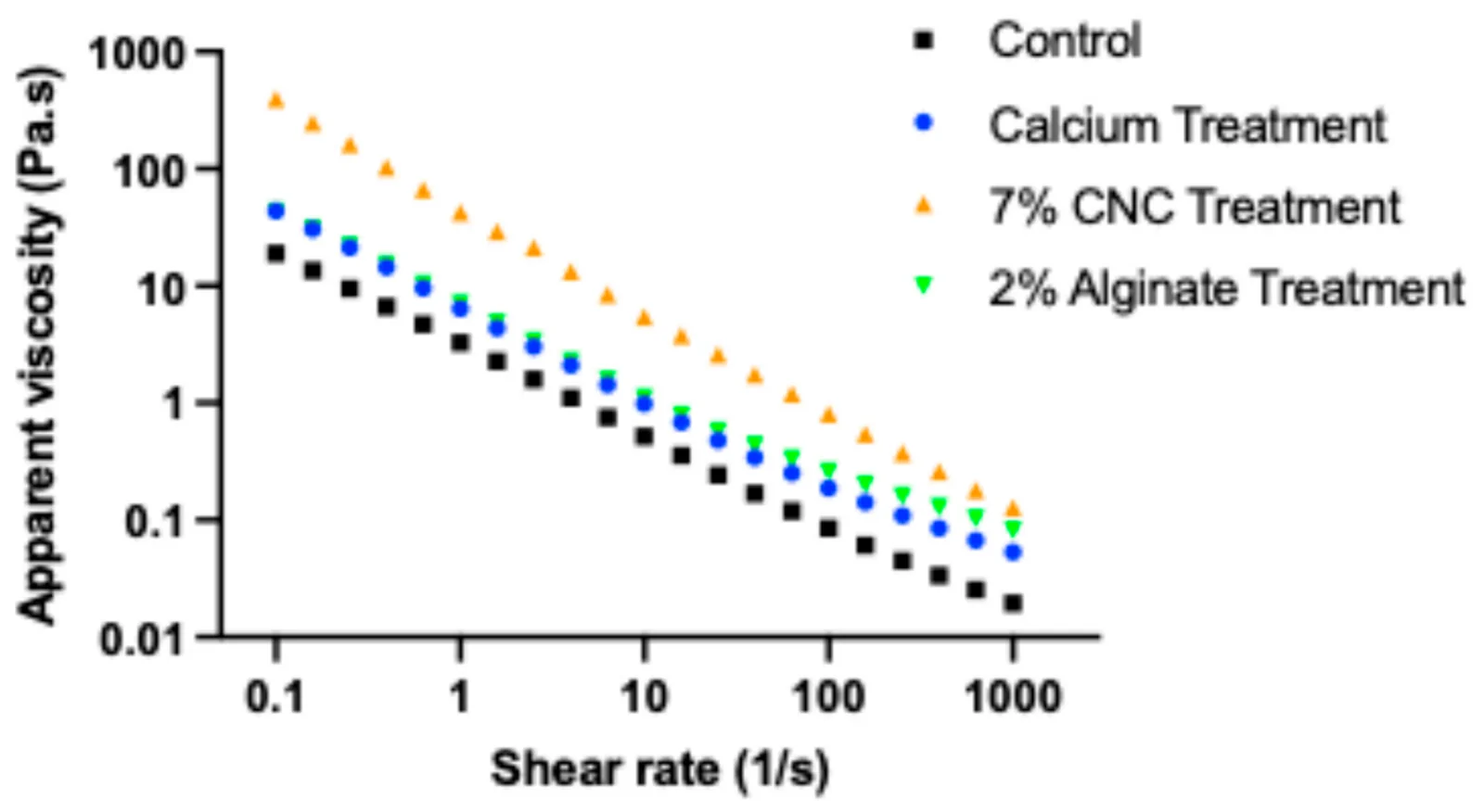
Apparent viscosity of mucus with various dietary treatments.

To further understand the interaction between mucus and dietary factors (calcium, CNC, and alginate), the rheology of mucus incorporating these dietary components was tested to provide a representative indication of local interactions at the fiber–mucus interface. The mechanical modulus and apparent viscosity of the mixture are shown in Figure 11 and Figure 12.

All samples exhibited elastic-dominant behavior (G′ > G″), indicating the capability of the mucus to resist deformation and preserve structural integrity under physiological shear during digestion. Calcium, CNC, and alginate increased the mechanical moduli of the mucus. Especially, 6% CNC group showed the greatest enhancement of mucus elasticity, indicating marked strengthening of the crosslinked gel network and improved resistance to deformation induced by luminal flow, which was consistent with the reduced mucus permeability observed in the CNC group. Similarly, Madsen et al. [57] reported a pronounced increase in the mechanical moduli when mucus was mixed with sodium carboxymethylcellulose, suggesting the fortified mucus gel structure induced by intermolecular interactions between the polymer and mucus.

As for the apparent viscosity, all samples exhibited pseudoplastic behavior. With increasing shear stress, individual mucin chains and those entangled with polysaccharides gradually disentangled because of the disruption of weak intermolecular interactions such as hydrogen bonding and electrostatic forces. This reduced polymer dimensions and increased mucus fluidity, resulting in a decrease in viscosity [15]. The incorporation of dietary calcium and soluble fibers increased the apparent viscosity of mucus, which may be associated with the interaction mechanisms described previously. Calcium can bind to the mucin protein to form reversible cross-links, generating linear or branched aggregates that are more structured than simple entanglements, which increases viscosity and strengthens the barrier against particle diffusion [16]. Alginate did not substantially alter mucus macroviscosity at the low concentration used in this study, suggesting that its barrier-enhancing effect may instead be associated with modulation of the mucus microenvironment, as described previously [53]. In contrast, CNC greatly increased mucus apparent viscosity, indicating potential bioadhesive interactions. Similarly, Lin, Shatkin and Kong [9] also reported rheological synergism between CNC and mucus, reflected by the enhanced apparent viscosity, which suggests strong intermolecular interactions between these two polymers.

The rheological results indicated that calcium and soluble fibers reinforced the mucus barrier through different biointeractive mechanisms. The resulting alterations in mucus rheology may, in turn, influence its barrier and lubrication functions in the GI tract.

## 4. Conclusions

In this study, the DGMS was successfully developed to evaluate nutrient permeability under dynamic digestion conditions with improved biorelevance and reproducibility compared with traditional static models. By incorporating dynamic luminal conditions together with a more physiologically relevant geometry, the DGMS provides a useful preliminary screening tool for evaluating nutrient and particle mucopenetration.

Using the DGMS, purified mucus showed compromised barrier function compared with native mucus, highlighting the importance of mucus preparation in permeability testing. Calcium affected nutrient permeability in a size-dependent manner, with larger molecules being more sensitive to calcium-induced changes in the mucus mesh. Dietary fibers, including alginate and CNC, also reduced glucose permeability through different mechanisms of mucus reinforcement. Overall, these findings demonstrate the utility of the DGMS for biorelevant mucus permeability assessment and provide insight into how dietary components may modulate nutrient transport through the mucus barrier.

## Figures and Tables

**Table 1:** The impact of luminal flow on glucose mucus permeability.

Models	P_app_ of Glucose (10^−6^ cm/s)
Static model	3.58 ± 0.19 **
DGMS	7.12 ± 0.59

Notes: DGMS, dynamic gastrointestinal mucus simulator; P_app_, apparent permeability coefficient. Values are presented as mean ± SD. Statistical significance was determined by independent two-tailed Student’s *t*-test. (**) *p* < 0.01 between DGMS and static mucus model.

**Table 2:** The impact of the nutrient molecular size on mucus permeability and thickness.

Nutrients	P_app_ (10^−6^ cm/s)	Mucus Thickness (mm)	Endpoint Images
Phycocyanin	0.74 ± 0.16	0.82 ± 0.06	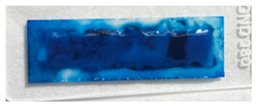
Glucose	7.12 ± 0.59 **	0.94 ± 0.10	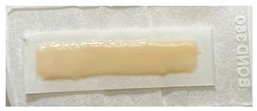

Note: Values are presented as mean values. Statistical significance was determined by two-tailed Student’s *t*-test. (**) *p* < 0.01 between permeability of different luminal molecules.

**Table 3:** Effects of luminal flow rate on glucose permeability and mucus thickness.

Flow Rate (mL/min)	P_app_ (10^−6^ cm/s)	Mucus Thickness (mm)	Endpoint Images
2.5	10.32 ± 0.42	0.86 ± 0.08	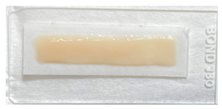
1	7.12 ± 0.59 **	0.94 ± 0.10	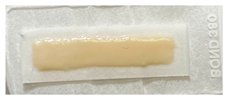

Note: Values are presented as mean values. Statistical significance was determined by the two-tailed Student’s *t*-test. (**) *p* < 0.01 between the permeability at different flow rates.

**Table 4:** Effects of mucus purification on glucose permeability and mucus thickness.

Mucus Type	P_app_ (10^−6^ cm/s)	Mucus Thickness (mm)	Endpoint Images
Native mucus	7.12 ± 0.59	0.94 ± 0.10	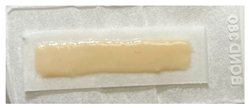
Purified mucus	58.34 ± 6.10 **	0.80 ± 0.09	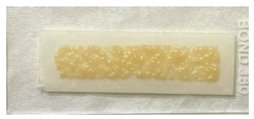

Note: Values are presented as mean ± SD. Statistical significance was determined by two-tailed Student’s *t*-test. (**) *p* < 0.01 between native mucus and purified mucus using the dynamic gastrointestinal mucus simulator (DGMS).

## Data Availability

The original contributions presented in this study are included in the article/Supplementary Material. Further inquiries can be directed to the corresponding author.

## Supplementary Materials

The following supporting information can be downloaded at https://www.mdpi.com/article/10.3390/foods15173084/s1: Figure S1: Representative images of mucus samples at the end of the permeability test.
