# Supplementary material for: A Dynamic Gastrointestinal Mucus Simulator for Biorelevant Assessment of Nutrient Mucopenetration: Effects of Mucus Purification, Calcium, and Soluble Fibers
Source: Foods. 2026 Aug 31;15(17):3084. doi: 10.3390/foods15173084 (PMC13565474; doi:10.3390/foods15173084)
Supplement: Supplementary file 1 [file foods-15-03084-s001.zip › foods-4497984-supplementary.pdf]

Supplementally Material

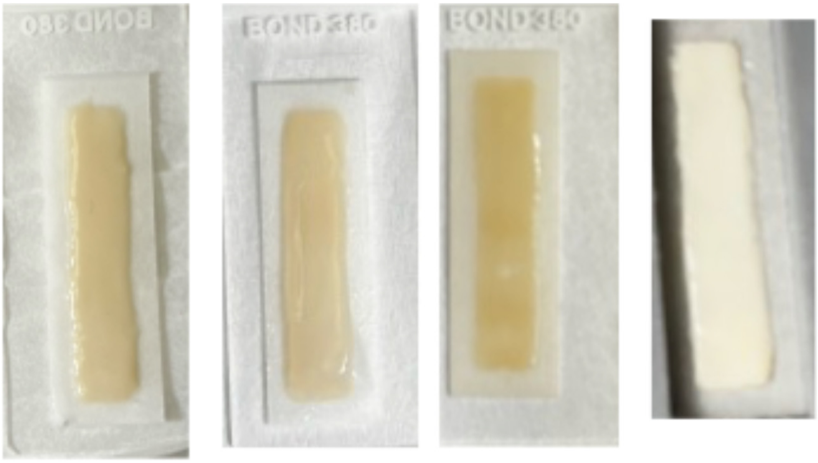

Figure S1. Representative images of mucus samples at the end of the permeability test. From left to right: native mucus, CNC-treated mucus (6%), alginate-treated mucus (2%), and calcium-treated mucus.
